# Predictors of Mediastinitis Risk after Coronary Artery Bypass Surgery: Applicability of Score in 1.322 Cases

**DOI:** 10.5935/abc.20170119

**Published:** 2017-09

**Authors:** Fabiana dos Santos Oliveira, Letícia Delfino Oliveira de Freitas, Eneida Rejane Rabelo-Silva, Laura Maggi da Costa, Renato Abdala Karam Kalil, Maria Antonieta Pereira de Moraes

**Affiliations:** Instituto de Cardiologia / Fundação Universitária de Cardiologia (IC/FUC), Porto Alegre, RS - Brazil

**Keywords:** Mediastinitis, Myocardial Revascularization / complications, Risk Management, Cohort Studies

## Abstract

**Background:**

Mediastinitis is a severe surgical complication of low incidence, but high lethality. Scores used in the preoperative period to stratify the risk of postoperative mediastinitis may contribute to improve the results.

**Objective:**

To test the applicability of the MagedanzSCORE in predicting the risk factors for mediastinitis in patients undergoing coronary artery bypass grafting at a cardiology reference hospital.

**Methods:**

Historical cohort study with adult patients who underwent coronary artery bypass grafting. The analyzed variables were contemplated in the MagedanzSCORE: reoperation, chronic obstructive pulmonary disease (COPD), obesity, class IV unstable angina, polytransfusion therapy, mediastinitis and death as outcome variables.

**Results:**

Of the 1.322 patients examined, 56 (4.2%) developed mediastinitis. Of these, 26 (46.4%) were classified as high risk for mediastinitis and 15 (26.8%) at very high risk for mediastinitis. Three of the five variables of the Magendanz Score showed statistically significant differences: reoperation, COPD and obesity. Class IV unstable angina and postoperative polytransfusion were not associated with mediastinitis after coronary artery by-pass grafting. The area under the ROC curve was 0.80 (CI 95% 0.73 - 0.86), indicating the model's satisfactory ability to predict the occurrence of mediastinitis.

**Conclusion:**

The tool was useful in the preoperative assessment demonstrating the risk for mediastinitis in this population of intensive care patients.

## Introduction

Mediastinitis is characterized as a deep infection of the surgical wound of heart surgery, with involvement of the retrosternal space, associated or not with sternal instability/osteomyelitis. The literature data suggest an incidence of 0.6 to 5.6% of this complication, with mortality rates between 14 and 32%, resulting in high rates of morbidity and mortality, extended duration of hospitalization, delayed postoperative recovery and increased hospital costs.^1-4^ In addition, it may be associated with a number of factors, including smoking, prolonged cardiopulmonary bypass (CPB) and the use of two mammary artery bypass.^5,6^

Estimating mediastinitis risks may contribute to the identification of potential complications in the preoperative period (PP), predicting in an individualized way which patients will need more intensive care, in order to develop preventive strategies.^6,7^ Previous studies point to tissue hypoperfusion, polytransfusion, impaired asepsis, surgical reintervention, infections and the use of antibiotic therapy as risk factors associated with a higher prevalence of mediastinitis during cardiac surgery.^8,9^

The use in clinical practice of tools that help decision making in the face of possible complications will certainly bring benefits to this population at greater risk. National authors developed a scoring model to predict the risk of mediastinitis in patients undergoing myocardial revascularization (CABG) surgery. Among the 2,809 patients evaluated, five variables were identified as independent predictors for the occurrence of mediastinitis: stable angina class IV/unstable angina (UA), chronic obstructive pulmonary disease (COPD), obesity, surgical reintervention and polytransfusion at the PP. The risk score proved to be easy to apply and directed to clinical practice.^10^

Careful clinical examination, associated with such instruments, allows health professionals to improve the identification of infection predictors during clinical evaluation. In view of the increasing number of cardiac surgeries, the high mortality rate in the occurrence of mediastinitis and the absence of data in our professional environment, it was developed this study with the objective of testing the applicability of MagedanzSCORE to predict the risk of mediastinitis in patients submitted to CABG in a reference hospital in cardiology in RS.

## Methods

### Design

Observational study of historical cohort.

### Population and sample

Study conducted with patients of both sexes, aged 18 years or older, submitted to isolated CABG, with or without CPB. Exclusion criteria were patients who had not recorded all the variables requested by the score. The convenience sample was estimated for variables predictors of infection. Considering a previous incidence of 1.0% in the institution, with OR of 3.5 of the surgical reintervention variable of the MagedanzSCORE, for a power of 80% and a level of statistical significance of 0.05, 1.322 patients were required.

### Study variables and outcomes

Data were collected through the review of medical records in the medical histories and entered in the database of the postoperative unit of cardiac surgery of the institution.

Demographic data, pre and trans-operative clinical data, antibiotic therapy and length of stay were analyzed. Score-related variables, such as surgical reoperation; COPD, clinically diagnosed and/or radiological study of the thorax and/or spirometry and/or drug treatment with corticosteroids and/or bronchodilator in the preoperative period; obesity (BMI ≥ 30 kg/m^2^); stable angina class IV or UA; polytransfusion (> 3 units of adult red cell concentrate at postoperative period). Outcomes analyzed included in-hospital mediastinitis (up to 30 days after surgery) and death from any cause, considered when it was after the date of diagnosis of Mediastinitis.

Mediastinitis was considered when it was diagnosed clinically or according to the criteria of the Centers for Disease Control and Prevention (CDC/NHSN),^11^ positive culture for pathogens of tissue or mediastinal fluid obtained during surgical intervention or needle aspiration; evidence of mediastinitis observed during surgical intervention or histopathological examination; patient with at least one of the following signs or symptoms with no other known cause: fever (body temperature> 38ºC), chest pain or sternal instability and at least one of the following: purulent discharge in the mediastinal area; organisms cultivated from blood or mediastinal area secretion; widening of the mediastinum on the X-ray. Superficial infection of the operative wound was not considered mediastinitis.

### Tested score

The instrument used was the MagedanzSCORE,^10^ prepared and validated previously.^12^ This is a predictive risk score for mediastinitis in patients undergoing CABG, composed of five variables predictors independent. The sum was classified into four groups: low risk (zero points), medium risk (1 to 2 points), high risk (3 to 4 points) and very high risk (≥ 5 points), according to chart 1.

**Chart 1:** MagedanzSCORE: prediction of risk for mediastinitis.

Clinical profile	Score
Surgical reoperation	3
COPD	2
Obesity	2
Class IV / unstable stable angina	1
Polytransfusion (post-operative)	1

### Ethical considerations and statistical analysis

This study was approved by the Ethics Committee in Research of the Institute of Cardiology - University Foundation of Cardiology of RS, under the number 4705/12. The Term of Commitment for the Use of Medical History Data was used.Data were analyzed through the Statistical Package for the Social Sciences (SPSS), version 22.0.

Categorical variables were expressed as absolute (n) and relative (%) frequencies and compared by the chi-square test. Continuous variables were expressed as mean ± standard deviation for those with normal or median distribution and interquartile range.

The performance of the MagedanzSCORE was evaluated by comparing the rate of mediastinitis presumed by the score with the one observed. To measure the discriminant power of the score, the area under the ROC curve was estimated. We used the multivariate analysis of the categorical variables to obtain the odds ratio (OR) and confidence interval (CI) with a significance level p < 0.05.

## Results

1.322 patients subjected to isolated CABG were included in this study, 84.5% performed a combined saphenous vein graft with two mammary artery grafts, and 97.4% used CPB. The mean age was 62.4 ± 9.8 years, and 72.6% of the patients were male.

The most prevalent independent predictors for mediastinitis were class IV/unstable angina (58.8%), followed by obesity (25.4%). The characteristics of the population are mentioned in Table 1.

**Table 1:** Characteristics of the population (n = 1322). Porto Alegre-RS

Characteristics	n (%)
Male	960 (72,6)
Age (years) *	62,4 ± 9,8
Body mass index (kg / m2) *	27,6 ± 4,2
Use of CPB 1.288	1.288 (97,4)
Three bypass grafting	696 (52,6)
Use of saphenous and double mammary artery bypass	1.117 (84,5)
Use of antibiotic therapy in the postoperative period	506 (38,3)
Preoperative hospitalization time (days) §	7 (0 – 69)
Total length of stay (days)§	41 (7 – 184)
**MagedanzSCORE (predictor variables)**	
Surgical reoperation	73 (5,5)
COPD	59 (4,5)
Obesity	336 (25,4)
Angina class IV / unstable	777 (58,8)
Polytransfusion (postoperative)	48 (3,6)
Mediastinitis	56 (4,2)
Death	7 (0,5)

* Data presented in mean ± standard deviation;

§ Data presented in median and intermediate. CPB: extracorporeal circulation; COPD: chronic obstructive pulmonary disease.

### Classification of risk and presence of mediastinitis according to MagedanzSCORE

**Figure 1:**
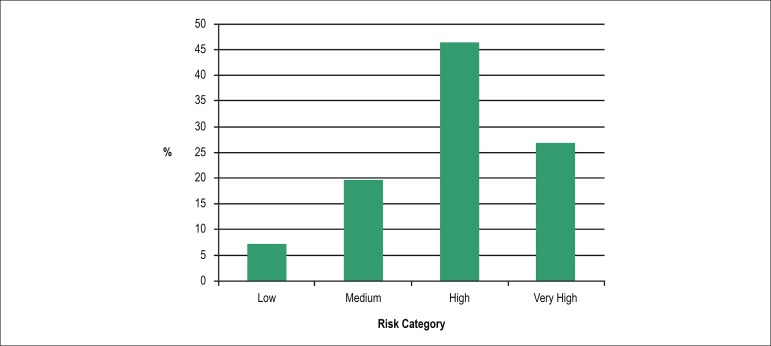
Presence of mediastinitis according to MagedanzSCORE. N = 56.

The risk of mediastinitis according to the MagedanzSCORE and the classification of that risk identified that 384 (29.1%) patients presented low risk, 651 (49.3%) medium risk, 256 (19.4%) high risk and 30 (2.3%) patients were classified with very high risk of developing the outcome (Figure 1).

Fifty-six (4.2%) patients developed mediastinitis after CABG. Of these, 26 (46.4%) were classified as having high risk and 15 (26.8%) with very high risk. Table 2 shows the distribution of the patients who presented the outcome, according to the MagedanzSCORE.

**Table 2:** Association between demographic variables and MagedanzSCORE in the occurrence of mediastinitis (multivariate analysis). Porto Alegre-RS

Variables	OR	IC 95%	p
Sex	1,76	0,92 – 3,31	0,085
Age	0,98	0,95 – 1,02	0,382
Surgical reoperation	37,76	18,75 – 77,92	< 0,001
COPD	3,83	1,23 – 10,46	< 0,001
Obesity	2,71	1,42 – 5,16	< 0,001
Angina class IV / unstable	1,88	0,95 – 3,96	0,072
Polytransfusion (postoperative)	0,51	0,15 – 1,52	0,236

COPD: chronic obstructive pulmonary disease; OR: odds ratio; 95% CI: 95% confidence interval

It was evidenced that three of the five variables predictive of infection presented statistically significant associations among them, namely, surgical reoperation, COPD and obesity. Demographic variables gender and age, as well as class IV / unstable angina and postoperative polytransfusion were not associated with mediastinitis after CABG.

**Figure 2:**
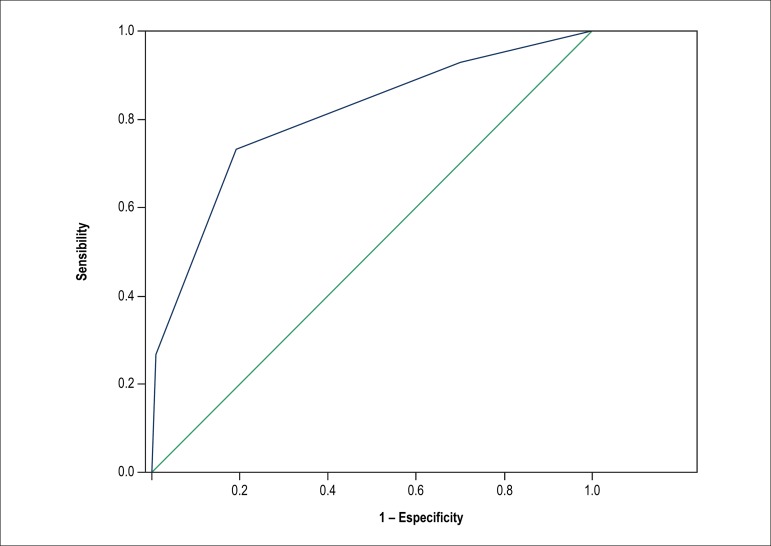
Area over the ROC curve in the measurement of the occurrence of mediastinitis. C: area under ROC curve; 95% CI: 95% confidence interval; 0.80 (95% CI 0.73-0.87).

The area under the ROC curve, used to measure the discriminant power of the score, was 0.80 (95% CI 0.73-0.87), demonstrating the model's satisfactory ability to predict the occurrence of mediastinitis at CABG isolated PP (Figure 2), compared to the validation study of the score^10^ that had accuracy measured by the area under the ROC curve of 0.73 (95% CI 0.68-0.80).

## Discussion

The results showed that the MagedanzSCORE is applicable and satisfactory to predict the risk of mediastinitis in this population of patients subjected to CABG. The applicability of risk scores in cardiac surgery is quite relevant, however it must be well evaluated, based on the real world population, so as not to underestimate or overestimate possible hospital events.^13,14^

The incidence of mediastinitis in this population was 4.2%, a value within the limits described in the literature, between 0.6 and 5.6%,^1,2^ although higher than the 3.3% published in the study that originated the score.^10^ An important result was that 73.2% of the patients who developed mediastinitis were classified in the high and very high risk groups. Similar results were found in the study that validated the instrument.^12^ These findings reinforce the effectiveness of the score to predict the outcome.

Patients with Class IV or unstable angina and obese constituted a large proportion of the sample studied, and each of these variables contributed with 1 and 2 points, respectively, to the risk score. A German study evaluating 1.700 similar patients found a strong association of obesity with infection, reinforcing that each increase of one kilogram of body mass per square meter produces a 3% increase in the risk of developing mediastinitis.^7^ The pathological mechanism involved in the association between obesity and mediastinitis is not yet well established. A previous study suggests that factors such as inadequate distribution of antibiotics due to increased body mass, difficult skin preparation, and large amounts of adipose tissue serving as a substrate for surgical wound infections may be involved in the mechanism of that association.^15^

Surgical reintervention is considered a predictor factor for the development of infections.^3,8,9^ In the present study, 5.5% of patients required reoperation and presented statistical significance when associated with the occurrence of mediastinitis. In a study that sought to identify similar risk factors in diabetics undergoing CABG, surgical reintervention was also relevant as a variable associated with increased risk.^12^

Another equally important predictor of risk among the results of this investigation was the presence of COPD, which had a strong association with the occurrence of mediastinitis, presenting statistical significance. Similar findings were described in previous investigations.^2,6,9^ Another study has cited COPD as a risk factor but not as an independent one. Patients with COPD would be more vulnerable to surgical wound infection due to tissues hypoxemia, and the use of corticosteroids at the pre and / or postoperative period would be a factor that could facilitate the installation of infectious processes.^15^

Class IV stable angina / unstable angina and postoperative polytransfusion, although independent predictors for the development of mediastinitis, were less important when they were associated with the outcome in this casuistic, although previous studies have linked these variables with increased risk of infection and morbidity at PP.^16,17^ In contrast to these findings, other authors found lower death rates in patients with UA undergoing isolated CA compared to those considered stable, attributing those results to possibly receiving better drug therapy, invasive monitoring and hemodynamic support with greater frequency.^18^

In this population studied, transfusion was not predictive of complication or worsening of results. However, data from the literature indicate that blood transfusions in the PP have been a constant concern, often reported as a contributing factor for infectious episodes, such as mediastinitis.^2,9,19^ Previous studies corroborate these data and reinforce that the number of units of concentrate of red blood cells in the PP is directly associated with an increased risk of complications.^10,20^

It is known that diabetes mellitus (DM) may difficult the recovery of patients undergoing cardiovascular surgeries; however, in this study this was not evaluated, because DM was not an independent predictor of risk for mediastinitis among the population that originated the score. We assume that these findings, considering the rigorous glycemic control and the continuous insulin use, could have collaborated for a satisfactory prognosis.

Many factors have been associated with the development of mediastinitis following cardiovascular surgery; however, there is no consensus in the literature about which are the most important and how much each represents as an independent predictor of high risk for mediastinitis.^10^ Other predictors cited in previous scores, such as age, sex and combined procedures, were evaluated and the five most important predictors for the development of MagedanzSCORE were defined.

Finally, the findings of this study allow us to infer that the instrument tested in the local population serves as a basis to aid in clinical practice. The limitations of the present study are those that are due to its retrospective nature and the search for medical records. Another factor worth highlighting is that the study has been developed in a single center specialized in cardiology, other studies are necessary to corroborate our observations, in order to disseminate the use of the score in clinical practice.

## Conclusion

Results showed that the score tested was applicable and satisfactory to predict the risk of mediastinitis in patients undergoing CABG at this institution. It could be incorporated into clinical practice as a useful tool to help identify risk predictors for the development of nfection in this more intensive care population.
